# Rosiglitazone: can meta-analysis accurately estimate excess cardiovascular risk given the available data? Re-analysis of randomized trials using various methodologic approaches

**DOI:** 10.1186/1756-0500-2-5

**Published:** 2009-01-10

**Authors:** Jan O Friedrich, Joseph Beyene, Neill KJ Adhikari

**Affiliations:** 1 Department of Medicine, University of Toronto, Toronto, Canada; 2 Interdepartmental Division of Critical Care, University of Toronto, Toronto, Canada; 3 Critical Care and Medicine Departments, and The Keenan Research Centre in the Li Ka Shing Knowledge Institute, St. Michael's Hospital, Toronto, Canada; 4 Dalla Lana School of Public Health, University of Toronto, Toronto, Canada; 5 Child Health Evaluative Sciences, Hospital for Sick Children Research Institute, Toronto, Canada; 6 Department of Critical Care Medicine and Sunnybrook Research Institute, Sunnybrook Health Sciences Centre, Toronto, Canada

## Abstract

**Background:**

A recent and provocative meta-analysis, based on few outcome events, suggested that rosiglitazone increased cardiovascular mortality and myocardial infarction. However, results of meta-analyses of trials with sparse events, often performed when examining uncommon adverse effects due to common therapies, can vary substantially depending on methodologic decisions. The objective of this study was to assess the robustness of the rosiglitazone results by using alternative reasonable methodologic approaches and by analyzing additional related outcomes.

**Findings:**

In duplicate and independently, we abstracted all myocardial and cerebrovascular ischemic events from all randomized controlled trials listed on the manufacturer's web site meeting inclusion criteria of the original meta-analysis (at least 24 weeks of rosiglitazone exposure in the intervention group and any control group without rosiglitazone). We performed meta-analyses of these data under different methodologic conditions. An unconfounded comparison that includes only trials (or arms of trials) in which medications apart from rosiglitazone are identical suggests higher risks than previously reported, making even the risk of cardiovascular death statistically significant. Alternatively, meta-analysis that includes all trials comparing a treatment arm receiving rosiglitazone to any control arm without rosiglitazone (as in the original meta-analysis) but also including trials with no events in both the rosiglitazone and control arms (not incorporated in the original meta-analysis), shows adverse but non-statistically significant effects of rosiglitazone on myocardial infarction and cardiovascular mortality. Rosiglitazone appears to have inconsistent effects on a wider range of cardiovascular outcomes. It increases the risk of a broad range of myocardial ischemic events (not just myocardial infarction). However, its effect on cerebrovascular ischemic events suggests benefit, although far from statistically significant.

**Conclusion:**

We have shown that alternative reasonable methodological approaches to the rosiglitazone meta-analysis can yield increased or decreased risks that are either statistically significant or not significant at the p = 0.05 level for both myocardial infarction and cardiovascular death. Completion of ongoing trials may help to generate more accurate estimates of rosiglitazone's effect on cardiovascular outcomes. However, given that almost all point estimates suggest harm rather than benefit and the availability of alternative agents, the use of rosiglitazone may greatly decline prior to more definitive safety data being generated.

## Background

A recent [1] and provocative [2-4] meta-analysis suggested that rosiglitazone increased cardiovascular morbidity and mortality. Given the popularity of this medication, the elevated risks of myocardial infarction (MI) (Peto odds ratio [OR] 1.43; 95% confidence interval [CI] 1.03–1.98; p = 0.03) and cardiovascular death (OR 1.64; 95% CI 0.98–2.74; p = 0.06) had broad public health implications. When interim results are added from the subsequently published RECORD trial [5], a phase III trial investigating cardiovascular outcomes, the effect on cardiovascular death is not significant (OR 1.08; 95% CI 0.78–1.51; p = 0.64) but the increased risk of MI remains (OR 1.35; 95% CI 1.04–1.75; p = 0.02). Other meta-analyses conducted by rosiglitazone's manufacturer [6], the US Food and Drug Administration [7], and the Cochrane Collaboration [8], in addition to two additional independent meta-analyses [9,10], supported these findings [1].

Meta-analysts [1] and others [2] have highlighted limitations, such as the short duration of many included trials that were not designed to assess cardiovascular outcomes, leading to potential ascertainment bias, in addition to sparse outcome events [3]. Indeed, results of a meta-analysis of trials with sparse events can vary substantially depending on methodologic and statistical decisions [11-13]. The objective of this study was to assess the robustness of the rosiglitazone results using alternative reasonable methodologic approaches and analyzing additional related outcomes. In particular, we examine four relevant issues not evaluated in previous analyses: 1) the effect of including only trials (or arms of trials) with an unconfounded comparison, in which medications apart from rosiglitazone are identical in the arms being compared, 2) the effect of using odds ratios, which can be biased when events are infrequent and group sizes are imbalanced, 3) the effect of including trials with no events in both groups, which would reduce pooled estimates of rosglitazone's effect, and 4) the effect of various definitions of ischemia outcomes (to examine for consistency of effect).

## Methods

All randomized controlled trials on the manufacturer's website [14] meeting inclusion criteria (any control group and at least 24 weeks of drug exposure) for the original meta-analysis [1] were reviewed. Our aim was to abstract data from the same database of clinical trials searched in the original meta-analysis [1], in addition to adding interim data from the RECORD trial published in response to the original meta-analysis. In duplicate, we selected trials from the database and abstracted data from included trials.

From each included trial, we abstracted data (number of patients in intervention and control arm with an outcome event and total patients in each arm) on the outcomes of myocardial infarction and cardiovascular death.  For the outcome of myocardial ischemia, we included all the following events (each was reported in at least one trial):  myocardial infarction (154 patients), myocardial ischemia (301 patients), new angina (44 patients), angina (36 patients), aggravated angina (19 patients), unstable angina (8 patients), cardiac chest pain (1 patient), coronary artery insufficiency (2 patients), revascularization (62 patients), coronary thrombosis (6 patients), coronary artery stenosis (2 patients), coronary artery occlusion (2 patients), coronary artery disorder (15 patients), coronary artery disease (7 patients), coronary artery atherosclerosis (2 patients), coronary artery spasm (1 patient), and three vessel disease (1 patient).  For the outcome of cerebrovascular event we included all the following events (each was reported in at least one trial):  stroke (64 patients), cerebrovascular accident (5 patients), cerebral infarction (1 patient), cerebral embolism (1 patient), cerebrovascular disorder (43 patients), transient ischemic attack (31 patients), cerebral ischemia (1 patient), vestibulobasilar insufficiency (1 patient), and carotid stenosis (2 patients).  For both myocardial ischemia and cerebrovascular events, trials did not specify which patients had more than one event among those listed above.  Therefore, if a trial reported outcome events in more than one category, in our primary analysis we assumed that the number of patients with an event was equal to the maximum number of patients in any one category.  In a sensitivity analysis we used the least conservative approach and assumed that each adverse event occurred in a different patient; our results did not change.

Interim data for RECORD, which was not included in the manufacturer's website [14], was obtained from reference 5 (myocardial infarction and cardiovascular death) and reference 7 (stroke: 29 strokes in 2220 rosiglitazone patients and 38 strokes in 2227 control patients). Data on other myocardial and cerebrovascular ischemic events were not available for RECORD.

Binary effect measure meta-analyses were carried out using standard equations and confirmed with Review Manager 4.2 (Cochrane Collaboration, Oxford, England) where possible. Odds ratios derived using exact statistical methods were calculated with StatXact 8 (Cytel Inc, Cambridge, MA, USA) and odds ratios derived using Bayesian methods were calculated with WinBUGS 1.4.1 [available at ]).

## Results

### Influence of Trial Selection Criteria

**Figure 1:**
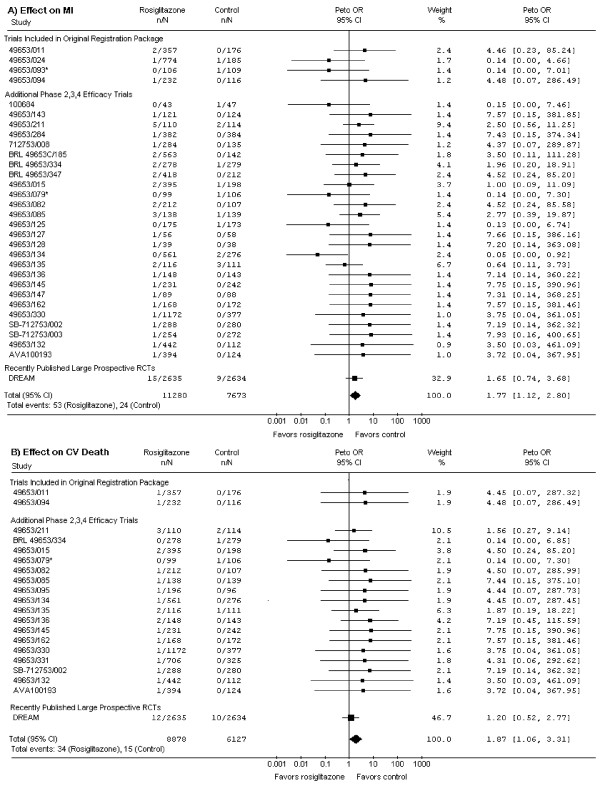
**Effects of Rosiglitazone on Myocardial Infarction (MI, panel A) and Cardiovascular (CV) Death (panel B)**. This analysis includes only trials in which the sole difference between the intervention and control groups is rosiglitazone therapy. Compared to the original analysis [1], this excludes trials AVM100264, 49653/020, 49653/080, 49653/097, 49653/137, SB-712753/009, and ADOPT; it also excludes RECORD. For the 3-armed trials (49653/079, 49653/093, and SB-712753/007 [marked with an '*']), we include only the treatment and control arms differing by rosiglitazone alone. Trials are ordered and grouped as per Table 1 in the original meta-analysis [1]. Only trials with events are shown. Weight refers to the contribution of each study's odds ratio (OR) to the overall pooled OR. The center of the diamond indicates the pooled OR, and the width of the diamond reflects the size of the 95% confidence interval (CI). Abbreviations: CI – confidence interval; CV – cardiovascular; MI – myocardial infarction; n – number of patients with event in the intervention or control group; N – total number of patients in the intervention or control group; OR – odds ratio; RCT – randomised controlled trial.

The original meta-analysis [1] included some randomized controlled trials in which the intervention and control groups did not differ only in the use of rosiglitazone. Some trials compared rosiglitazone to active control, and in several 3-arm trials, groups receiving rosiglitazone alone and groups receiving rosiglitazone and other hypoglycemic agents were combined into 1 treatment group in the meta-analysis. (RECORD also compared rosiglitazone to active control.) In such trials, assessment of the risk of rosiglitazone is confounded. Including only trials (or arms of trials) in which medications apart from rosiglitazone are identical should provide a more specific estimate of rosiglitazone's effect. Such a meta-analysis (Figure 1) suggests an even higher risk of MI (p = 0.01) and cardiovascular death (p = 0.03).

### Influence of Effect Measure

**Figure 2:**
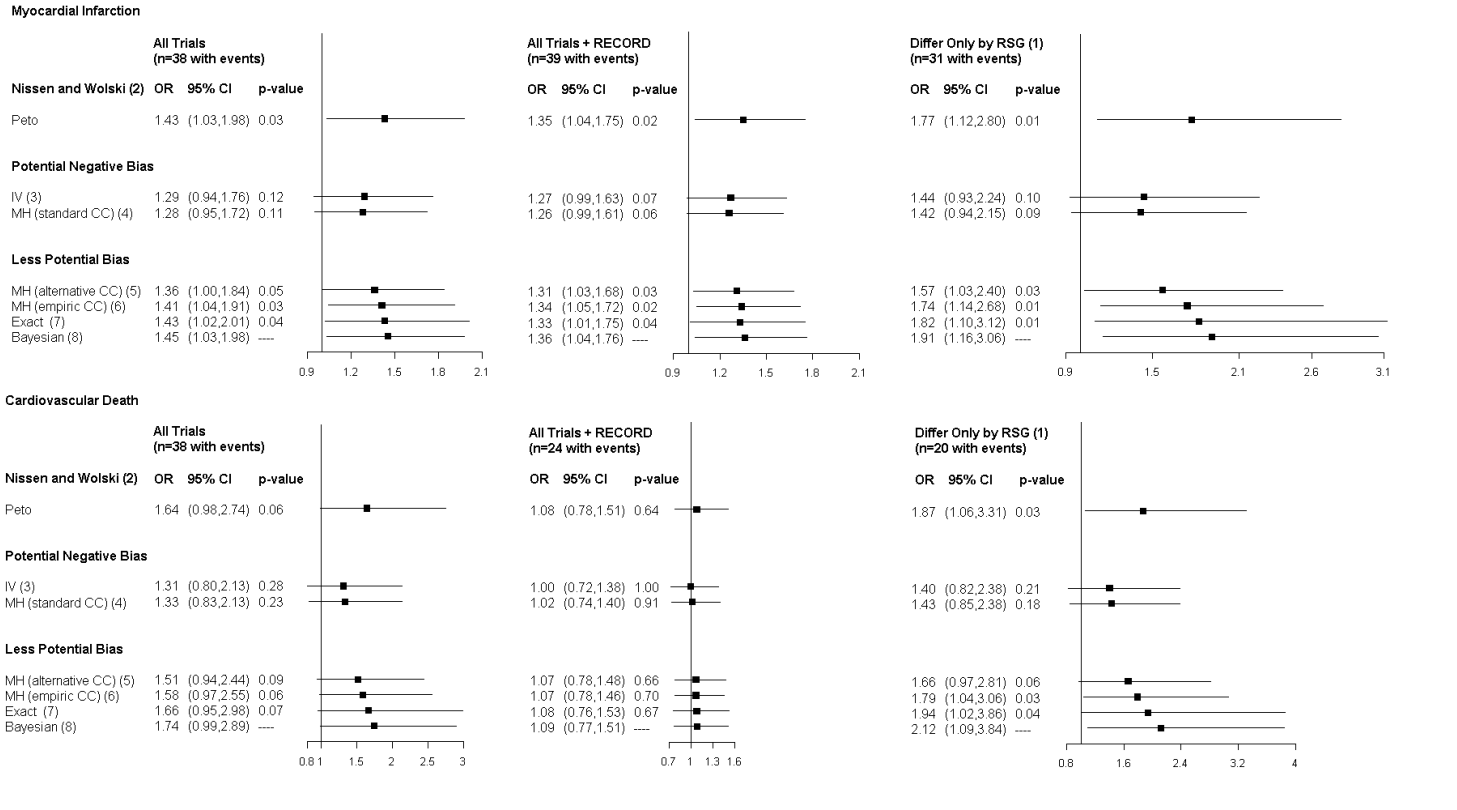
**Effect of Rosiglitazone on Myocardial Infarction and Cardiovascular Mortality**. For each adverse clinical outcome, the pooled effect of rosiglitazone in different trial groupings is expressed as odds ratios (OR) and 95% confidence intervals (CI) using different statistical methods. For the Bayesian OR, CI denotes credible interval. Statistical methods with "Potential Negative Bias" may show reduced treatment effects. (1) This analysis includes only trials in which the sole difference between the intervention and control groups is rosiglitazone therapy. Compared to the original analysis, this excludes trials AVM100264, 49653/020, 49653/080, 49653/097, 49653/137, SB-712753/009, and ADOPT. It excludes one arm in each of the 3-armed trials 49653/093, 49653/079, and SB-712753/007. It also excludes RECORD. (2) Peto Odds Ratios as reported by Nissen and Wolski [1]. (3) Inverse Variance (IV) Odds Ratios using standard 0.5 continuity corrections in trials with no events in one group (Review Manager Software using a Random Effects Model with no heterogeneity [*I*^2 ^= 0]). (4) Mantel-Haenszel (MH) Odds Ratios using standard 0.5 continuity corrections (CC) in trials with no events in one group (Review Manager Software using a Fixed Effects Model). (5) Mantel-Haenszel (MH) Odds Ratios using treatment arm continuity corrections (CC) in trials with no events in one group as proposed by Sweeting et al [11] to minimize bias. (6) Mantel-Haenszel (MH) Odds Ratios using empiric continuity corrections (CC) in trials with no events in one group as proposed by Sweeting et al [11] to minimize bias. (7) Odds Ratios calculated using exact statistical methods with no requirement for continuity corrections (StatXact 8 [Cytel Inc, Cambridge, MA, USA]). (8) Odds Ratios along with 95% credible intervals calculated using fixed effects Bayesian method and non-informative prior distributions with no requirement for continuity corrections (WinBUGS 1.4.1 [available at ]). Abbreviations: CC – continuity corrections; CI – Confidence or Credible Interval; *I*^2 ^– *I*^2 ^heterogeneity statistic; IV – inverse variance; MH – Mantel Haenszel; OR – Odds Ratio; RSG – rosiglitazone.

The overall event rates in the rosiglitazone trials, even including RECORD, are extremely low (0.7% for MI and 0.4% for cardiovascular death). Simulation studies [11,12] have shown that in such circumstances, commonly used pooled odds ratios using inverse variance or Mantel-Haenszel methods are negatively biased, showing reduced treatment effects. Indeed, analyses using these methods show *no *statistically significant effects, with most p-values ≥ 0.10 (Figure 2). Although the authors of the original meta-analysis [1] used Peto OR to minimize this bias, many trials had imbalanced group sizes. Simulation [11,12] also suggests that even Peto OR may underestimate true effect sizes under these conditions [15]. Using other odds ratio methods less prone to bias (such as Mantel-Haenszel methods employing continuity corrections that minimize bias, exact statistical methods, and Bayesian methods with non-informative priors), the risk of adverse events was similar (Figure 2), suggesting that Peto OR is an acceptable effect measure for these data despite important theoretical shortcomings.

### Influence of the Exclusion of Trials with No Outcome Events

Regardless of the specific form of odds ratio, however, this effect measure forces the exclusion of studies with zero events in both the intervention and control arms [16]. This occurs because odds ratios from such studies become undefined due to division by zero when standard methods are used [13]. In addition to the 42 trials in the original meta-analysis [1], we identified an additional 12 trials on the manufacturer's web site [14] meeting inclusion criteria but with no myocardial infarctions (though some listed other myocardial ischemic or cerebrovascular events) and no deaths from cardiovascular causes in both intervention and control arms. Evaluating all 54 trials, 16 trials with 4241 patients (30% of all trials; 14% of 30242 patients in all trials) recorded no myocardial infarctions and 31 trials with 9801 patients (57% of trials; 32% of 30242 patients) recorded no cardiovascular deaths.

**Figure 3:**
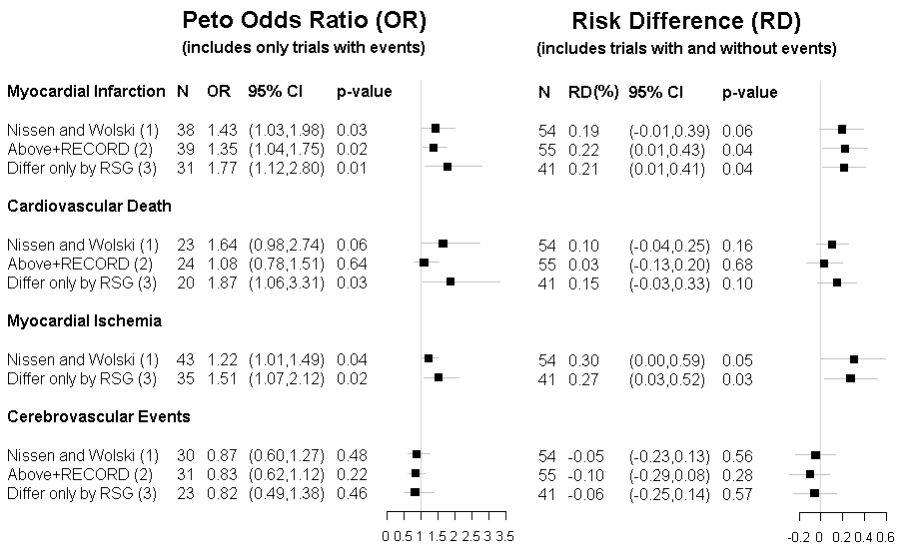
**Effect of Rosiglitazone on Adverse Clinical Outcomes**. For each adverse clinical outcome, the pooled effect of rosiglitazone in different trial groupings is shown using Peto OR (which includes trials with events) and RD (which includes trials both with and without events).    The RD analysis includes trials with 24 or more weeks of drug exposure listed on the manufacturer’s web site () meeting the inclusion criteria of the original meta-analysis [1]. In addition to trials included in the original meta-analysis [1], we also include 6 additional trials with an active control group  (BRL 49653/282, BRL 49653/369, BRL 49653/392, BRL 49653/207, BRL 49653/325, 49653/109), 5 additional trials comparing rosiglitazone to placebo (49653/044, 49653/096, BRL 49653/131, 49653/390, 49653/452), and one additional 4-armed trial with some arms differing only by rosiglitazone therapy (SB-797620/004).  For myocardial ischemia and cerebrovascular morbidity individual trials did not specify which patients had more than one adverse event.  Consequently,if there were events in more than one category, we took the most conservative approach that the least number of patients in a particular trial arm had all these adverse events.  Alternatively, using the least conservative approach by assuming that each adverse event occurred in a different patient, the cerebrovascular result did not significantly change and the p-value for the myocardial ischemia results decreased. (1) Nissen and Wolski refers to the original meta-analysis [1]. (2) This analysis includes data from all trials in the original meta-analysis [1] in addition to the RECORD trial [5]. (3) This analysis includes only trials in which the sole difference between the intervention and control groups is rosiglitazone therapy.  Compared to the original analysis, this excludes trials AVM100264, 49653/020, 49653/080, 49653/097, 49653/137, SB-712753/009, and ADOPT.  It excludes one arm in each of the 3-armed trials 49653/093, 49653/079, and SB-712753/007.  It also excludes RECORD. Abbreviations:  CI – confidence interval; N – number of trials included in each meta-analysis; OR – Odds Ratio; RD – Risk Difference; RSG – rosiglitazone.

The inclusion of such zero total event trials would move effect estimates closer to nil. In principle, including such trials is possible for any effect measure [13], but standard methods allow it only for risk difference (RD). Re-analysis of the data from all 54 trials using Mantel-Haenszel RD, which is less prone to bias than inverse variance RD in low event rate situations [12], shows no significant increase in myocardial infarction (p = 0.06) or cardiovascular death (p = 0.16) (Figure 3). If one includes only trials (or arms of trials) in which medications other than rosiglitazone are identical, the risk difference for MI just retains significance at the p = 0.05 level (Figure 3). The increased absolute risk of MI of 0.21% (95% CI 0.01%–0.41%), if true, implies a number needed to harm of 476 (95% CI 244–10,000) patients.

### Consistency of Effect across Related Outcomes and over Time

Clinicians and regulators grappling with the implications of important but subtle distinctions in meta-analysis methodology may look to corollary evidence to guide decisions regarding rosiglitazone. One approach to examine the robustness and consistency of the myocardial infarction results would be to analyze rosiglitazone's effect on *all *events resulting from myocardial ischemia. Doing so and using the same statistical methods also suggests increased risk (Figure 3). A related approach would be to examine whether rosiglitazone's effect is similar in different vascular territories. When the same methods are used for cerebrovascular ischemic events, there is no evidence of harm and, in fact, some suggestion of benefit (Figure 3). Thus, there appears to be consistency of effect using various definitions of cardiac morbidity, but a lack of consistency between cardiac and cerebrovascular effects. This difference may reflect unknown mechanisms of rosiglitazone's effects on different vascular territories (for example, increased congestive heart failure may contribute to increased myocardial ischemia with little effect on cerebral ischemia) or may simply highlight chance differences in trial outcomes when absolute event rates are low.

**Figure 4:**
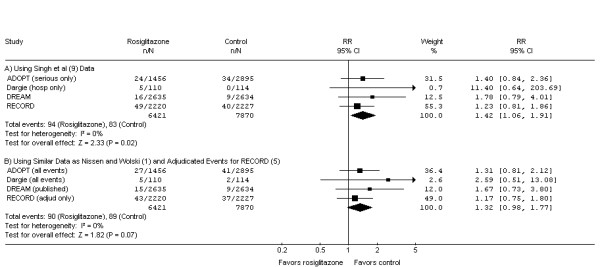
**Effect of rosiglitazone on myocardial infarction (MI) in randomized controlled trials with at least 12 months of follow-up**. Singh et al [9] included only "serious events" rather than "total events" for ADOPT [17], only events leading to hospitalization rather than all investigator-reported MIs for Dargie et al [19], and for the DREAM study [18] included an additional rosiglitazone patient reported to have an MI that was included in a recent presentation by the manufacturer to the Food and Drug Administration [7] but not included in the original publication. In contrast, Nissen and Wolski [1] included all MIs for ADOPT [17] and Dargie et al [19] and used the published values for DREAM [18]. While the decisions made by Singh et al [9] were reasonable, each increased the risk of MI for rosiglitazone relative to control and thus contributed to a statistically significant result (Panel A). Alternatively, using the same event inclusion decisions as Nissen and Wolski [1] and including only adjudicated events from the subsequently published RECORD trial [5], the increase in MI no longer retains significance at the p = 0.05 level (Panel B). If one limits the analysis to the two long-term trials in which the intervention and control groups differ only by rosiglitazone therapy [18,19] the risk increases; however, again it loses its statistical significance when the data used by Singh et al (RR 2.27 [95%CI 1.06 to 4.87], p = 0.03) are replaced by those used by Nissen and Wolski (RR 1.83 [95%CI 0.88 to 3.81], p = 0.11). For this figure we have used the same effect measure as Singh et al [9] (Risk Ratio [RR]); the p-values are identical if Peto OR is used as the effect measure. Weight refers to the contribution of each study's RR to the overall pooled RR. The center of the diamond indicates the pooled RR, and the width of the diamond reflects the size of the 95% CI. Abbreviations: CI – confidence interval; *I*^2 ^– *I*^2 ^heterogeneity statistic; MI – myocardial infarction; n – number of patients with event in the intervention or control group; N – total number of patients in the intervention or control group; OR – odds ratio; RR – risk ratio; Z – Z test statistic.

Finally, assessment of longer-term outcomes may support the interpretation of results based on short-term follow-up data. A recent meta-analysis [9] including the 4 published trials with at least 12 months of follow-up [5,17-19] also reported a statistically significant increased risk of MI (p = 0.02). However, compared to the original meta-analysis [1], these authors [9] included slightly different numbers of myocardial infarction events for each of the 3 trials [17-19] common to the two papers. If instead they had used the same event inclusion criteria as the original meta-analysis [1] and included only adjudicated events from RECORD [5], they would not have found a statistically significant effect of rosiglitazone on MI (p = 0.07) (Figure 4).

## Discussion

Inferences regarding rosiglitazone's effect on adverse cardiovascular events, based on meta-analysis of available randomized controlled trials studying primarily low-risk patients for relatively short intervals, depend on selection of trials and effect measure. We have demonstrated that different reasonable methodological approaches can yield increased or decreased risks that are either statistically significant or not significant at the p = 0.05 level for both MI and cardiovascular death. Overall, our new analyses support concerns regarding increased cardiovascular risks of rosiglitazone. However, they also highlight the challenge of attributing rare adverse events to a particular drug and the importance of using sensitivity analyses when results are of borderline statistical significance.

We examined four methodologic issues relevant to a meta-analysis of rosiglitazone's effects that were not completely considered in previous meta-analyses [1,2,6-10]. First, we determined the overall unconfounded effect of rosiglitazone by comparing groups differing only by rosiglitazone therapy and showed higher estimates of harm that achieve statistical significance even for cardiovascular death. Second, we used other less biased effect measures for low event rate data and found similar effects. Third, we explored consistency of effects across outcomes; using a broader definition of myocardial ischemia events we also found statistically significant harm, but we did not find an increased risk of cerebrovascular events. Finally, we included additional trials with no outcome events, an approach which decreases the magnitude of treatment effects, and found that all risk difference point estimates for rosiglitazone still suggest harm rather than benefit, although some p-values are not statistically significant. Others [2] have also re-calculated inverse variance and Mantel-Haenszel odds ratios by including zero total event trials (using methods suggested by us [13]) and showed reduced estimates of harm due to rosiglitazone. However, their results [2] are likely strongly influenced by the bias towards no effect exhibited by inverse variance and Mantel-Haenszel odds ratios when event rates are very low. In contrast, our analyses including zero total event trials used an effect measure that minimized this bias to evaluate the impact of such trials for rosiglitazone.

## Conclusion

In summary, our additional new analyses strengthen the original results, which have been questioned because of sparse outcome events and borderline statistical significance [3]. Ideally, a large randomized trial adequately powered to detect differences in infrequent cardiovascular adverse events would be conducted, especially since results from such trials often differ from meta-analyses of smaller trials [20]. However, given the very small absolute increase in event rates, such a trial would need to be extremely large. Ongoing [5] and future clinical trials may help to generate more accurate estimates of rosiglitazone's effect on cardiovascular outcomes, but will need to study higher-risk patients for longer time periods, as rosiglitazone is intended for life-long use. Unfortunately, ongoing trials like RECORD may still be underpowered to provide definitive data due to lower-than expected events rates and other trial design issues [7].

Other related data support concerns about rosiglitazone's effects. A population-based observational study [21], despite also having relatively few events and a study design more prone to confounding, also suggests increased cardiovascular risk from rosiglitazone. In addition, another recent meta-analysis [22] without the same statistical issues suggests that pioglitazone, the other commercially available thiazolidinedione, is not associated with increased cardiovascular morbidity. The statistical issues discussed here are less important for the pioglitazone meta-analysis because the event rates are higher and the proportion of zero event rate trials is probably lower (the exact proportion of such trials, considering both included and excluded trials in the denominator, cannot be calculated from data available in the paper [22]). Moreover, the largest trial, a phase III trial, with a mean follow up of almost 3 years [23], which compared pioglitazone to placebo, dominated the results. This trial almost demonstrated a statistically significant benefit in the primary endpoint that included all-cause mortality, non-fatal myocardial infarction, and stroke, among others, on its own. Thus, data from these related studies makes an adequately powered trial simply to disprove harm difficult to justify from both ethical (potential harm to patients) and resource perspectives.

Given the growing population with type 2 diabetes and their high prevalence of cardiovascular disease, the accurate and reliable determination of *any *cardiovascular risks and benefits has enormous public health implications. However, because available clinical trial data for rosiglitazone do not suggest improved clinical outcomes and *possible *harm, and because alternative agents are available, clinicians may simply stop prescribing rosiglitazone without definitive safety conclusions ever being generated. Although such a situation is not ideal, given the available imperfect data, such a course of action does not appear unreasonable.

## Abbreviations

CI: confidence interval; MI: myocardial infarction; OR: odds ratio; RD: risk difference.

## Competing interests

The authors declare that they have no competing interests.

## Authors' contributions

JOF and NKJA were involved in the design of the study, data acquisition, data analysis and interpretation, drafting of the manuscript, and critical review of the manuscript for important intellectual content. JB was involved in the design of the study, data analysis and interpretation, and critical review of the manuscript for important intellectual content. All authors have read and approved the final version of the manuscript.

## Response

By Sonal Singh^1 ^and Yoon K. Loke^2^

Email: sosingh@wfubmc.edu

Address: ^1^Wake Forest University School of Medicine, Winston-Salem, NC 27157, USA.

^2^University of East Anglia, Norwich NR4 7TJ, UK.

This report by Friedrich *et al*. utilizes various meta-analytic techniques to measure the cardiovascular risk of rosiglitazone. Irrespective of the methodology used, the results are the same. They report a similar increased risk of myocardial infarction (and cardiovascular death-statistically significant at times) as seen in our rigorous meta-analysis of long term trials with rosiglitazone, which showed that rosiglitazone is associated with increased ischemic risk with long term use in patients with type 2 diabetes as compared to alternative therapies [9].

However, there are several challenges in measuring rare but important adverse effects in randomized trials because of incomplete reporting of outcomes and inconsistent definitions. The short term trials may be inadequately powered to detect long term adverse effects, and hence we focused on the long term trials. We used the latest version of the published data, and consistently extracted similar categories of events such as those requiring hospitalization or classified as serious adverse events, irrespective of whether adjudicated or not because different trials may have different ways of adjudication [9]. The use of adjudicated and non-adjudicated events may give different risk estimates. We also provided sensitivity estimates based on the adjudicated events which showed a similar increased risk [9].

The inclusion of zero event trials as done by the authors of this article is problematic as it is unclear if no events were reported or whether events did not occur. Several of these problems can be fixed by consistent reporting of outcomes of all trials and methodological consensus on how best to conduct meta-analysis on sparse events.

However the authors overemphasize the precise estimate of the RRs and the corresponding P values, which are likely to vary given the different inclusion and exclusion criteria. More important than the absolute precisions of risk, the consistency of effects across comparisons suggests a higher cardiovascular risk with rosiglitazone compared to placebo or active controls, despite the modest benefit in glycemic control seen with rosiglitazone. Finally observational studies have also proved to be useful and shown similar increased cardiovascular risk with rosiglitazone [21,24].

Hence accurate estimates of cardiac risk on ongoing trials are unlikely to change this general scientific consensus recommendations by the American Diabetes Association that *" rosiglitazone be avoided in the treatment of type 2 diabetes *[25]. Infact, one can argue that it would be unethical to conduct such a trial, and expose patients to harm, to measure the precise cardiovascular risk of rosiglitazone, given the overwhelming existing evidence on the cardiovascular adverse effects of rosiglitazone [26,27]. Unfortunately a drug used to treat type 2 diabetes, where 2/3^rd ^of deaths are due to cardiovascular death, should at the minimum be cardiac neutral, which is not the case with rosiglitazone which raises the risk of cardiovascular adverse effects no matter what methodology is used.

Competing interests: None
